# Compromised Rat Testicular Antioxidant Defence System by Hypothyroidism before Puberty

**DOI:** 10.1155/2012/637825

**Published:** 2012-01-16

**Authors:** Dipak K. Sahoo, Anita Roy

**Affiliations:** ^1^Departments of Zoology and Biotechnology, Utkal University, Bhubaneswar, 751004 Orissa, India; ^2^KTRDC, College of Agriculture, University of Kentucky, Lexington, KY 40546-0236, USA

## Abstract

Altered thyroid function during early stages of development is known to affect adversely testicular growth, physiology, and antioxidant defence status at adulthood. The objective of the present study is to investigate the modulation of antioxidant defence status in neonatal persistent hypothyroid rats before their sexual maturation and also to identify the specific testicular cell populations vulnerable to degeneration during neonatal hypothyroidism in immature rats. Hypothyroidism was induced in neonates by feeding the lactating mother with 0.05% 6-n-propyl-2-thiouracil (PTU) through the drinking water. From the day of parturition till weaning (25 day postpartum), the pups received PTU through mother's milk (or) drinking water and then directly from drinking water containing PTU for the remaining period of experimentation. On the 31st day postpartum, the animals were sacrificed for the study. An altered antioxidant defence system marked by elevated SOD, CAT, and GR activities, with decreased GPx and GST activities were observed along with increased protein carbonylation, disturbed redox status in hypothyroid immature rat testis. This compromised testicular antioxidant status might have contributed to poor growth and development by affecting the spermatogenesis and steroidogenesis in rats before puberty as indicated by reduced germ cell number, complete absence of round spermatids, decreased seminiferous tubule diameter, and decreased testosterone level.

## 1. Introduction

Most vertebrates are unable to grow and reach their normal adult form without the thyroid hormone [1, 2]. The testis was considered to be a thyroid hormone unresponsive tissue [3]. However, various subsequent studies revealed that thyroid hormone plays a key role in rat testis development [4–7]. Mammalian testis is a target of thyroid hormone action and altered thyroid function which is known to affect testicular functions [8, 9].

Thyroid hormone is well known as a physiological modulator of oxidative stress [10, 11]. Previous studies on the role of thyroid hormone in testis are mainly focused on histological and physiological aspects resulting into reproductive failure [6, 8, 12–14]. Thyroid hormone exerts major influences on the developing testes [15]. Altered thyroid function during early stages of development and maturation may adversely affect testicular growth and physiology [7–9]; particularly the Sertoli cells, which play a major role in spermatogenesis and are the main cell type in the testis which expresses T_3_ receptors [16]. Studies indicate that there is an increase in T_3_ receptors in the neonates [5]. It is well established that the formation of normal numbers of Sertoli cells is a key factor in determining testis size, germ cell numbers per testis, and sperm production rate in adulthood in a range of mammals, including humans [17].

In our earlier reports, it was demonstrated that experimentally hypothyroidism modulates several oxidative stress and antioxidant defence parameters in mitochondria and postmitochondrial fractions in adult rat testis [18, 19]. We also reported about the effects of transient and persistent hypothyroidism on testicular antioxidant defence system in mature rats to know the role of hypothyroidism-induced oxidative stress in testicular development and maturation [7].

However, information about the role of deprivation of thyroid hormone in early developmental growth and function of testis in relation to antioxidant status in immature rats is inadequate. In rat, the onset of spermatogenesis occurs around 5 days of age. Spermatogenesis reaches the pachytene stage at 20 days of age. At 25 days of age, round spermatids appear, and at the age of 50 days, mature spermatozoa are released into seminiferous tubular lumen [20, 21]. Fetal type Leydig cells disappear soon after birth (during first 2 weeks after birth) and are replaced by adult type Leydig cells [22, 23]. Sertoli cell proliferation reaches its maximum level just before birth and ceases by the age of 3 weeks [24]. Hence, four weeks or around 30 days of age, testis is undergoing critical stages of development and thyroid hormone is playing a key role during this stage. In our earlier studies, it was reported that transient hypothyroidism (from day 1 of neonatal age till day 30) modulated testicular antioxidant defence status as well as functions in adult stage [7]. Moreover, it was also shown that germ cells in these rats were under oxidative stress and had poor antioxidant defence system [25]. So, it is interesting to know about the testicular antioxidant defence status and oxidative stress parameters along with testicular physiology at the 30 days of age, that is, before puberty.

The objective of the present study is to investigate the modulation of antioxidant defence status in neonatal persistent hypothyroid rats before their sexual maturation. In addition, we have tried to identify the specific cell populations in testes vulnerable to degeneration during neonatal hypothyroidism in immature rats.

## 2. Materials and Methods

### 2.1. Animals and Treatments

Male pups obtained from breeding were made hypothyroid from day 1 of neonatal age till day 30 of postnatal age. Hypothyroidism was induced in neonates by feeding the lactating mother with 0.05% 6-n-propyl-2-thiouracil (PTU) through the drinking water [7, 18, 25]. From the day of parturition till weaning (25 day postpartum), the pups received PTU through mother's milk (or) drinking water and then directly from drinking water containing 0.05% PTU for the remaining period of experimentation. Adult male Wistar rats (*Rattus norvegicus*) were divided into two groups containing 15 animals each. Group-I rats served as control for Group II rats. Group-II rats were treated with PTU from day 1 postpartum to day 30 postpartum. Animal care, maintenance, and experiments were conducted under the supervision of the Institutional Animal Ethics Committee (IAEC) regulated by the Committee for the Purpose of Control and Supervision of Experiments on Animals (CPCSEA), Government of India.

### 2.2. Sample Preparation and Hormone Estimations

On the 31st day postpartum, body weight of animals in Group-I and II was recorded; the animals were sacrificed by decapitation, trunk blood was collected and allowed to clot and then centrifuged to obtain sera. The serum levels of total T_3_, T_4_, TSH, and testosterone were measured by using ELISA kits (Monobind, Inc., Costa Mesa, CA, USA, and Equipar diagnostici, Italy). Testes and accessory sex organs, that is, seminal vesicle, ventral prostate and epididymis were removed, cleaned in cold 0.15 mol/L NaCl (normal saline), pat dried, and weighed. Testes were kept at −80°C till further use. Testes from three animals were pooled to one sample and five such samples were taken for the study.

### 2.3. Isolation of Mitochondrial and Postmitochondrial Fractions and Biochemical Analyses

The whole procedure of tissue processing was done as described earlier by Sahoo et al. (2008) [7]. In brief, a 20% (w/v) homogenate of testis was prepared in 50 mM phosphate buffer, PH 7.4, containing 0.25 M sucrose. The crude homogenate (CH) was centrifuged at 600 g for 10 min to precipitate nuclei and other cellular debris. The resulted supernatant was again centrifuged at 10,000 g for 20 min to separate mitochondria. The supernatant obtained was the postmitochondrial fraction (PMF). The mitochondrial pellet was washed thrice in 50 mM phosphate buffer, pH 7.4 (10,000 g for 5 min each), and finally suspended in the same buffer to obtain mitochondrial fraction (MF). For lipid peroxidation (LPx), tissue samples were processed as described above except using the buffer without sucrose. Protein content of samples was estimated using bovine serum albumin as standard [26].

### 2.4. Oxidative Damages to Lipids and Proteins

LPx was measured in CH and MF by monitoring the formation of thiobarbituric acid-reactive substances (TBARSs) following Ohkawa et al. (1979) [27]. The assay was performed in presence of 0.02% (w/v) butylated hydroxytoluene to suppress artefactual peroxidation during heating. Malondialdehyde (MDA) was used as the standard and TBARS were expressed in terms of MDA equivalents as nmol TBARS formed/mg protein. Protein carbonyl (PC) content was estimated in testicular CH, MF, and PMF following Levine et al. (1990) [28] and was expressed as nmol/mg protein.

For measuring protein-SH content, PMF and MF samples were first precipitated in ice-cold 5% trichloroacetic acid (TCA) containing 0.01 M HCl and centrifuged at 1000 ×g for 15 min. Protein precipitates dissolved in 8 M guanidine hydrochloride were then used to measure thiol content and presented as *μ*mol/mg protein [29, 30].

### 2.5. Reduced Glutathione (GSH), Oxidized Glutathione (GSSG), and H_2_O_2_ Content

Total glutathione content was measured in the supernatant of TCA-precipitated testicular PMF and MF samples by enzymatic recycling procedure and GSSG was measured after masking GSH with 2-vinylpyridine [31, 32]. GSH content was obtained from difference between these two values. Only GSH plus twice the GSSG levels were denoted as total GSH equivalents [7]. Total GSH equivalents, GSH and GSSG contents were expressed as nmol/g tissue. Hydrogen peroxide (H_2_O_2_) content was determined in testicular PMF and MF following horseradish peroxidase-dependent oxidation of phenol red [33]. H_2_O_2_ content was expressed as nmol/mg protein in testicular PMF or MF.

### 2.6. Antioxidant Enzyme Activities

For measuring the activity of catalase (CAT), PMF treated with ethanol (0.17 M) and Triton X-100 (1%) was directly used. The enzyme activity was measured following decomposition of H_2_O_2_ at 240 nm as described earlier [34] and expressed as nkat/mg protein. Superoxide dismutase (SOD) activity in PMF (for Cu/Zn-SOD) and MF (for Mn-SOD) of testis was determined following modified nitrite method [35]. One unit of enzyme activity was defined as the amount of enzyme capable of inhibiting 50% of nitrite formation under assay conditions.

Glutathione peroxidase (GPx) activity was assayed in PMF and MF by measuring oxidation rate of NADPH in presence of hydroperoxide, GSH, and glutathione reductase (GR) [19, 36]. Total and selenium-dependent GPx activities were estimated by using cumene and tert-butyl hydroperoxides, respectively. The difference between total glutathione peroxidase and selenium-dependent glutathione peroxidase (Se-D-GPx) activities represents the selenium-independent glutathione peroxidase (Se-I-GPx) activity [19, 37]. Glutathione reductase (GR) activity in testicular PMF and MF was assayed by measuring oxidation rate of NADPH in presence of GSSG [38]. In testicular PMF and MF, glutathione-S-transferase (GST) activity was measured following change in absorbance of the conjugated product of GSH and 1-chloro, 2, 4-dinitrobenzene (CDNB) at 340 nm [39]. GPx, GR, and GST enzyme activities were expressed as nkat/mg protein.

### 2.7. Glucose-6-phosphate Dehydrogenase

Glucose-6-phosphate dehydrogenase (G6PD) activity was assayed in PMF by measuring NADPH formation from glucose-6-phosphate along with NADP [40] and expressed as nkat/mg protein.

### 2.8. Histology

Following sacrifice, testis tissues were immediately fixed for histological studies in freshly prepared sublimate formal, dehydrated in graded ethanol series, cleared in xylene, and embedded in paraffin wax. Tissues were sectioned and stained with Hematoxylin and Eosin. The sections were observed under light microscope for qualitative and quantitative characterization. Numbers of germ cells and Sertoli cells were recorded as described earlier [7].

### 2.9. Statistics

All data represent means ± standard deviation and were subjected to unpaired Student's *t*-test to find out the level of significance between control and experimental rats. Minimal statistical significance was accepted at *P* < 0.05.

## 3. Results

### 3.1. Weights of Body, Testes, and Accessory Sex Organs

The weight gain in Group-II rats on 31st day of age was almost 50% less than Group-I control rats (Table 1). The weight of testis, seminal vesicle, and ventral prostate (g/100 g body wt) decreased significantly in PTU-treated rats of Group-II (Table 2).

### 3.2. Serum Hormone Profile

The serum T_3, _T_4,_ and testosterone level decreased significantly whereas the level of TSH increased by several folds in Group-II rats in comparison to control rats in Group-I (Table 3).

### 3.3. Histology, Germ Cell Count

Marked decrease in seminiferous tubule diameter was observed in neonatal persistent hypothyroid 30-day-old rats (Group-II) as compared to the corresponding controls (Table 4). There was a significant decrease in spermatogonia (24%) and spermatocytes (79%) accompanied by complete absence of round spermatids in Group-II testis (Table 4). However, a significant increase in the Sertoli cell number in Group-II rat testis was marked (Table 4).

### 3.4. Oxidative Stress Parameters

**Figure 1:**
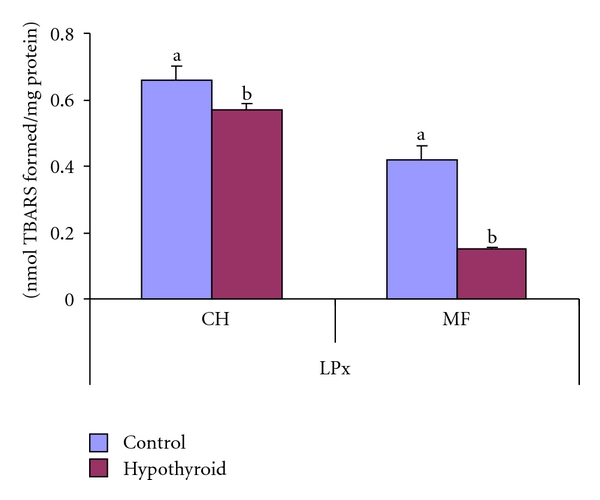
Effect of neonatal PTU treatment on lipid peroxidation (LPx; nmol TBARS formed/mg protein) in crude homogenate (CH) and mitochondrial fraction (MF) of testes of rats. Data are expressed as mean ± S.D. of 5 observations and subjected to unpaired Student's *t*-test. Statistical significance was accepted at *P* < 0.05. Control and hypothyroid groups were found to differ significantly at *P* < 0.05 as represented by superscripts of different letters. Control (Group-I): 30-day-old control rats; hypothyroid (Group-II): 30-day-old rats with PTU treatment from day 1 postpartum to day 30 postpartum.

The level of endogenous lipid peroxidation showed a decrease by 13.6% and 64% in crude and mitochondrial fractions, respectively, in response to 30 days persistent PTU treatment (group-II) in comparison to control (Group-I) rats (Figure 1).

**Figure 2:**
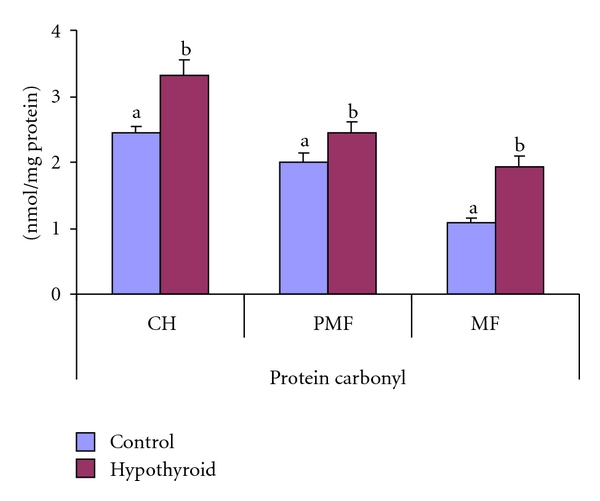
Effect of neonatal PTU treatment on protein carbonyl content (nmol/mg protein) in crude homogenate (CH), postmitochondrial fraction (PMF), and mitochondrial fraction (MF) of testes of rats. Data are expressed as mean ± S.D. of 5 observations and subjected to unpaired Student's *t*-test. Statistical significance was accepted at *P* < 0.05. Control and hypothyroid groups were found to differ significantly at *P* < 0.05 as represented by superscripts of different letters. Control (Group-I): 30-day-old control rats; hypothyroid (Group-II): 30-day-old rats with PTU treatment from day 1 postpartum to day 30 postpartum.

A significant elevation in protein carbonyl content was recorded in the crude homogenate (by 35.6%), mitochondrial (78%), and postmitochondrial fraction (22%) of testis of Group II hypothyroid rats when compared to the controls (group I, Figure 2).

**Figure 3:**
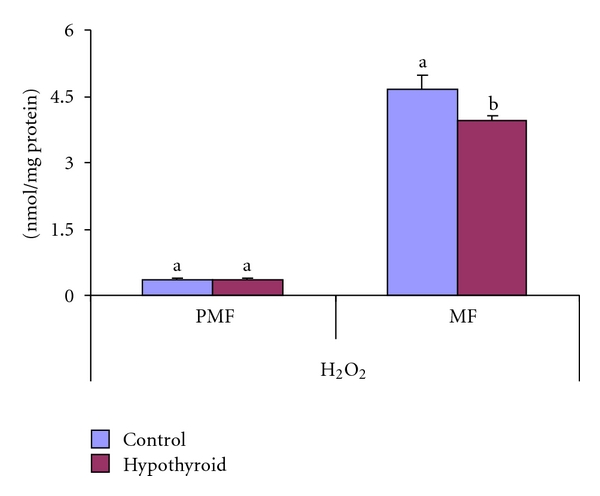
Effect of neonatal PTU treatment on hydrogen peroxide (H2O2; nmol/mg protein) in postmitochondrial fraction (PMF) and mitochondrial fraction (MF) of testes of rats. Data are expressed as mean ± S.D. of 5 observations and subjected to unpaired Student's *t*-test. Statistical significance was accepted at *P* < 0.05. Control and hypothyroid groups were found to differ significantly at *P* < 0.05 as represented by superscripts of different letters. Control (Group-I): 30-day-old control rats; hypothyroid (Group-II): 30-day-old rats with PTU treatment from day 1 postpartum to day 30 postpartum.

Hydrogen peroxide content remained unaltered in PMF and decreased (by 15%) in MF of Group-II PTU-treated rats (Figure 3). 

### 3.5. Protein-SH

**Figure 4:**
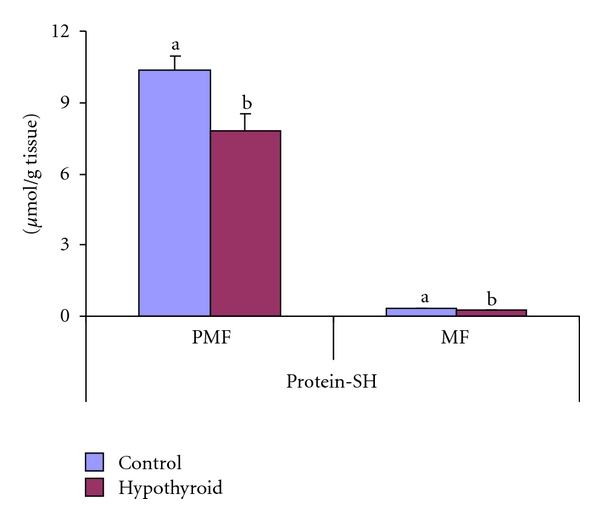
Effect of neonatal PTU treatment on protein-SH content (*μ*mol/g tissue) in postmitochondrial fraction (PMF) and mitochondrial fraction (MF) of testes of rats. Data are expressed as mean ± S.D. of 5 observations and subjected to unpaired Student's *t*-test. Statistical significance was accepted at *P* < 0.05. Control and hypothyroid groups were found to differ significantly at *P* < 0.05 as represented by superscripts of different letters. Control (Group-I): 30-day-old control rats; hypothyroid (Group-II): 30-day-old rats with PTU treatment from day 1 postpartum to day 30 postpartum.

The protein-SH content decreased significantly by 14% and 24%, respectively, in mitochondrial and postmitochondrial fractions of testis of Group-II rats (Figure 4). 

### 3.6. Total, Oxidized, and Reduced Glutathione Contents

In mitochondrial fraction of testis of group-II, rats, total GSH equivalent, oxidized, and reduced glutathione contents and GSH to GSSG ratio declined, respectively, by 50%, 16.7%, 67%, and 61% (Table 5). On the other hand, the Group-II testicular postmitochondrial total GSH equivalent and reduced glutathione contents did not alter with 23.5% decrease in GSSG activity and 27% elevation in GSH to GSSG ratio (Table 5). 

### 3.7. Antioxidant Enzyme Activities

**Figure 5:**
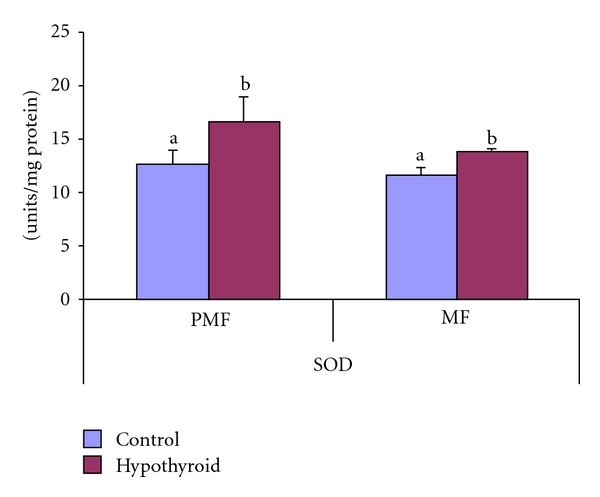
Effect of neonatal PTU treatment on superoxide dismutase activity (SOD; units/mg protein) in postmitochondrial fraction (PMF) and mitochondrial fraction (MF) of testes of rats. Data are expressed as mean ± S.D. of 5 observations and subjected to unpaired Student's *t*-test. Statistical significance was accepted at *P* < 0.05. Control and hypothyroid groups were found to differ significantly at *P* < 0.05 as represented by superscripts of different letters. Control (Group-I): 30-day-old control rats; hypothyroid (Group-II): 30-day-old rats with PTU treatment from day 1 postpartum to day 30 postpartum.

**Figure 6:**
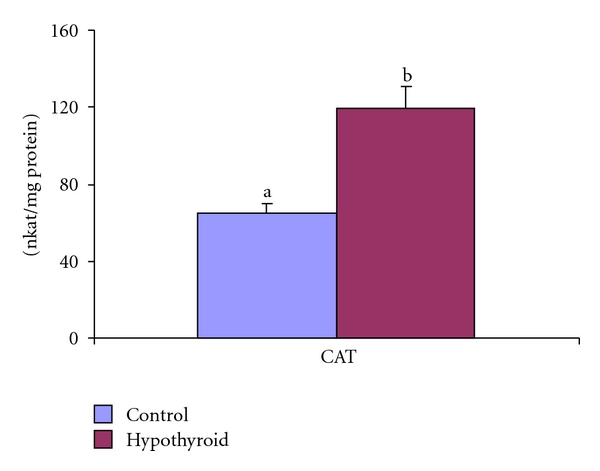
Effect of neonatal PTU treatment on catalase activity (CAT; nkat/mg protein) in postmitochondrial fraction (PMF) of testes of rats. Data are expressed as mean ± S.D. of 5 observations and subjected to unpaired Student's *t*-test. Statistical significance was accepted at *P* < 0.05. Control and hypothyroid groups were found to differ significantly at *P* < 0.05 as represented by superscripts of different letters. Control (Group-I): 30-day-old control rats; hypothyroid (Group-II): 30-day-old rats with PTU treatment from day 1 postpartum to day 30 postpartum.

The Mn-SOD activity was elevated by 20% in MF whereas Cu/Zn-SOD activity was increased by 30% in PMF of testis of Group-II rats (Figure 5). In the postmitochondrial fraction of testis, the CAT activity was increased by 83% in response to PTU treatment in Group-II rats in comparison to control rats (Group-I) (Figure 6).

**Figure 7:**
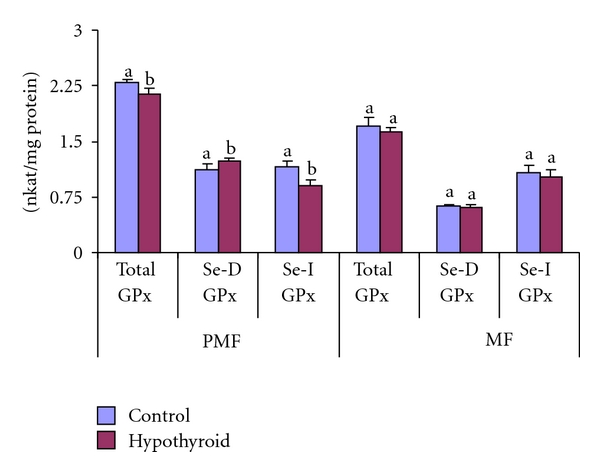
Effect of neonatal PTU treatment on glutathione peroxidase activity (GPx; nkat/mg protein) in postmitochondrial fraction (PMF) and mitochondrial fraction (MF) of testes of rats. Data are expressed as mean ± S.D. of 5 observations and subjected to unpaired Student's *t*-test. Statistical significance was accepted at *P* < 0.05. Control and hypothyroid groups were found to differ significantly at *P* < 0.05 as represented by superscripts of different letters. Control (Group-I): 30-day-old control rats; hypothyroid (Group-II): 30-day-old rats with PTU treatment from day 1 postpartum to day 30 postpartum.

**Figure 8:**
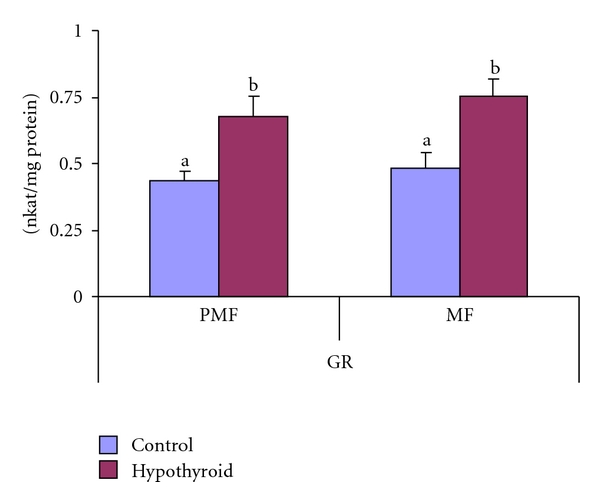
Effect of neonatal PTU treatment on glutathione reductase activity (GR; nkat/mg protein) in postmitochondrial fraction (PMF) and mitochondrial fraction (MF) of testes of rats. Data are expressed as mean ± S.D. of 5 observations and subjected to unpaired Student's *t*-test. Statistical significance was accepted at *P* < 0.05. Control and hypothyroid groups were found to differ significantly at *P* < 0.05 as represented by superscripts of different letters. Control (Group-I): 30-day-old control rats; hypothyroid (Group-II): 30-day-old rats with PTU treatment from day 1 postpartum to day 30 postpartum.

In Group-II rat testicular mitochondrial fraction, the GPx activities remained unaltered (Figure 7). However, in postmitochondrial fraction, total GPx and Se-I-GPx declined, respectively, by 6.7% and 22.35% with 9.5% elevation in Se-D-GPx activity (Figure 7). The GR activity increased significantly by 56% and 55%, respectively, in mitochondrial and postmitochondrial fractions of testis of Group-II rats in response to PTU treatment for 30 days (Figure 8).

**Figure 9:**
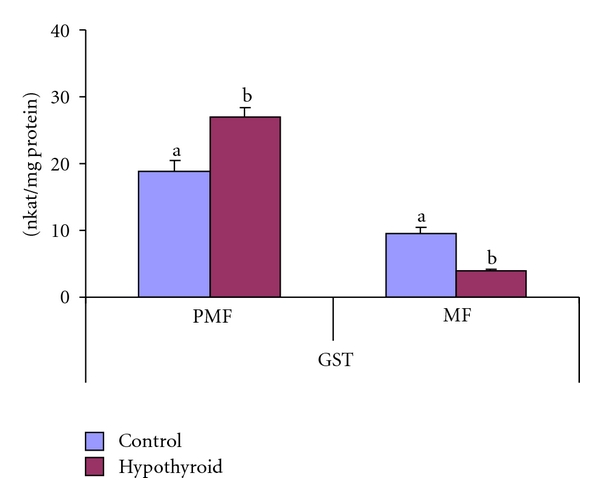
Effect of neonatal PTU treatment on glutathione S-transferase activity (GST; nkat/mg protein) in postmitochondrial fraction (PMF) and mitochondrial fraction (MF) of testes of rats. Data are expressed as mean ± S.D. of 5 observations and subjected to unpaired Student's *t*-test. Statistical significance was accepted at *P* < 0.05. Control and hypothyroid groups were found to differ significantly at *P* < 0.05 as represented by superscripts of different letters. Control (Group-I): 30-day-old control rats; hypothyroid (Group-II): 30-day-old rats with PTU treatment from day 1 postpartum to day 30 postpartum.

 The GST activity decreased by 59% in mitochondrial fraction and elevated by 42% in postmitochondrial fraction of testis in response to PTU treatment in Group-II rats when compared to control rats (Group-I, Figure 9).

### 3.8. Glucose-6-phosphate Dehydrogenase (G6PD)

**Figure 10:**
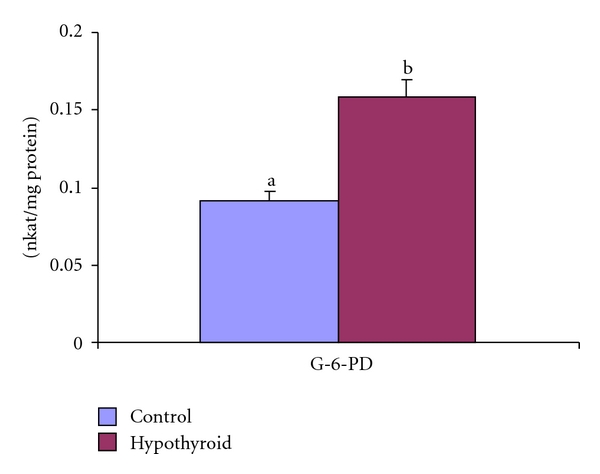
Effect of neonatal PTU treatment on Glucose-6-phosphate dehydrogenase activity (nkat/mg protein) in postmitochondrial fraction (PMF) of testes of rats. Data are expressed as mean ± S.D. of 5 observations and subjected to unpaired Student's *t*-test. Statistical significance was accepted at *P* < 0.05. Control and hypothyroid groups were found to differ significantly at *P* < 0.05 as represented by superscripts of different letters. Control (Group-I): 30-day-old control rats; hypothyroid (Group-II): 30-day-old rats with PTU treatment from day 1 postpartum to day 30 postpartum.

An increase in enzyme activity in post mitochondrial fraction of testis was recorded in Group-II rats by 73% (Figure 10).

## 4. Discussion

Thyroid hormones are reported to play a critical role in growth, differentiation, maturation, and metabolism in vertebrates [41]. Essential role of thyroid hormone in male sexual maturation, and reproduction was reported earlier [4, 6, 42]. Both hyperthyroidism and hypothyroidism states alter testicular antioxidant defence parameters in adult rats [7, 18, 19, 25, 43–45]. However, the effects of persistent hypothyroidism on neonatal rats before their puberty need to be studied. The present study reports the effect of neonatal persistent hypothyroidism induced by PTU treatment on the testicular antioxidant system and spermatogenic function in rats before puberty. The efficacy of the treatment was confirmed by a dip in T_3_ and T_4_ levels and a consequent increase in TSH level in serum of neonatal persistent hypothyroid rats. PTU is well known to decrease the conversion of peripheral T_4_ to T_3_ and thereby reduces serum T_3_ concentration. A significant reduction in the body weight was observed in PTU-treated rats when compared to control rats as reported earlier in case of neonatal hypothyroidism [46].

 The lower H_2_O_2_ contents in hypothyroid rats might be due to lower superoxide production in hypothyroid state due to hypometabolic rate. The hypothyroid rat testis resulted in a reduction in lipid peroxidation, which might be as a result of metabolic depression due to hypothyroid condition which serves as a protective factor to prevent lipid peroxidation [18, 47, 48]. In another study, Mogulkoc et al. (2005) [49] also observed a low lipid peroxide content in testicular and renal tissues of hypothyroid rats.

 On the contrary, oxidative stress is marked as the increase in protein carbonyl contents in crude and mitochondrial fraction of hypothyroid rat testis in the present study. It is in good agreement with Choudhury et al. (2003) [18] and Sahoo et al. (2008) [7], who observed an increased level of carbonylation of protein in crude homogenate of testes of hypothyroid rats.

Superoxide dismutase (SOD; EC 1.15.1.1) constitutes the first line of coordinated enzymatic defense against ROS by dismutating O_2_^•−^ into O_2_ and H_2_O_2_. Catalase (CAT; EC 1.11.1.6) and glutathione peroxidase (GPx; EC 1.11.1.9) are most crucial for detoxifying H_2_O_2_, thereby preventing the generation of hydroxyl radical by the Fenton reaction. Selenium-dependent glutathione peroxidases (Se-D GPxs) are the major selenoprotein-containing gene family in mammals [50]. Among the different types of selenium-dependent hydroperoxide-reducing isozymes, phospholipid hydroperoxide glutathione peroxidase (PH-GPx/GPx-4; EC 1.11.1.12) and classic cellular glutathione peroxidase (cGPX/GPx-1; EC 1.11.1.9) are primarily found in testis [19, 44, 51]. Selenium-dependent glutathione peroxidases contribute to a part of the total GPx activity. Other GPx activities in mammalian systems are selenium independent [52]. The Se-independent GPx (Se-I GPx), component of GST alfa class (accession: IPR003080 GST_alpha), is responsible for GPx activity in testis [53]. A significant elevation in total SOD and CAT activities in response to neonatal persistent hypothyroidism and a simultaneous decrease in total GPx, Se-D GPx (GPx-1 and GPx-4), and Se-I GPx in the PMF of testis in experimental group suggested that SOD and CAT have predominant role to fight oxidative stress than GPx in hypothyroid rats. The majority of the cytosolic GPx in rat testis existed as selenium- and nonselenium-dependent GPx which is present in the Leydig cells. Much lower levels are associated in sertoli and spermatogenetic cells [54]. GPx is primarily responsible for H_2_O_2_ removal in testicular mitochondria that does not contain catalase. GPx plays a crucial role in scavenging peroxyl radicals and thereby maintains functional integrity of the cell membrane, spermatogenesis, sperm morphology, and motility [55]. In testis, PH-GPx or GPx-4 which is partially cytosolic and partially bound to nuclei and mitochondria is localized within maturating spermatogenetic cells [54]. Gpx-1 has mitochondrial and cytosolic subcellular localizations in all mammalian tissues [56]. We observed that mitochondrial Se-dependent GPx (including GPx1 and GPx-4) activity was unaltered in hypothyroid rats as reported earlier by Chattopadhyay et al. (2010) [19]. In fact, the role of cGPx/GPx1 in protecting testes from oxidative injury is questionable as cGPx knockout mice were found to be fertile [57]. It has also been suggested that the metabolic pathway of testosterone biosynthesis requires protection against peroxidation and will be affected by a decrease in the GPx activity [58]. The lower serum testosterone level in hypothyroid rats in the present study also corroborates the fact.

 The decreased GSH level hypothyroid rat testis was recorded in the present study. Mammalian testis contains relatively high levels of GSH [55], which is reported to play an important role in the proliferation and differentiation of spermatogenic cells [59] by protecting them from noxious effects of ROS [60]. The level of GSH is different in testicular cell types, having maximum level in Sertoli and germ cells [61]. Thyroid hormone is known to play a role in triggering the biosynthesis of GSH [62], therefore under hypothyroid states, it is possible that there may be a fall in GSH levels contributing to susceptibility of the testes to oxidative stress. The ratio of reduced glutathione (GSH) to oxidized glutathione (GSSG) in tissues is also considered as one of the important marker of oxidative stress [63]. The decrease in its ratio and also a decrease in GSH contents in testicular MF of hypothyroid rats suggest severely hindered GSH metabolism. Such type of decreased GSH contents in testis-mitochondria of hypothyroid rats was also reported earlier [19]. Moreover, declined GSH contents in germ cells were reported earlier in case of transient hypothyroid rats [25]. The decreased protein-SH contents in both mitochondrial and postmitochondrial fractions might be due to the response against disturbed testicular redox pool in hypothyroidism. Similar effects on testicular protein-SH during hypothyroidism were reported by Chattopadhyay et al. (2010) [19]. 

 Glutathione-S-transferase (GST; EC 2.5.1.18) metabolizes xenobiotics by conjugating with GSH. In fact, GST-catalysed conjugation of GSH with exogenous compounds and endogenous metabolites such as 4-hydroxynonenal is regarded as major cellular defense mechanism against toxicity [64]. The decreased activity of this enzyme in MF of testes in neonatal persistent hypothyroid rats reflects the inability of the testis to counteract oxidative stress by this pathway. Glutathione reductase (GR; EC 1.6.4.2) is the enzyme responsible for maintenance of GSH by reducing GSSG back to GSH. Stimulated GR activity in neonatal hypothyroid rats indicated that mitochondrial metabolism attempted reduction of GSSG to GSH in order to maintain redox status. Such type of GR elevation was also reported in hypothyroid rat testis by Sahoo et al. (2008) [7] and Chattopadhyay et al. (2010) [19]. Increase in activity of glucose-6-phosphate dehydrogenase (G6PD) due to neonatal hypothyroidism might be due to higher demand for NADPH as GR requires this reducing equivalence to generate reduced GSH from GSSG. This fact is corroborated by increased GR activities in testicular postmitochondrial fraction too.

The testis seems to be the most vulnerable during neonatal persistent hypothyroidism as evident from the significantly decreased germ cell count. There were also almost no round spermatids found in the testis of hypothyroid rats. Simorangkir et al. (1997) [65] also showed impairment of spermatogenesis in hypothyroid rats. They reported absence of round spermatids in 30 days hypothyroid testis and the inability of the spermatogenic cells to complete meiosis. Francavilla et al. (1991) [66] also reported an impaired spermatogenesis and increased degeneration of germ cells in testis in response to prolonged hypothyroidism in rats. Increased Sertoli cell number in hypothyroid rats in the present investigation might be due to extended Sertoli cell proliferation as in an earlier report, Van Haaster et al. (1992) [67] and Holsberger and Cooke (2005) [68] demonstrated that neonatal hypothyroidism may increase adult Sertoli cell populations by extending Sertoli cell proliferation.

The serum testosterone levels were significantly reduced in the hypothyroid group. As hypothyroidism causes significant decrease in LH and FSH with a fall in serum testosterone [69, 70], this might lead to a disruption of the spermatogenic and steroidogenic processes. Moreover, it has been reported that neonatal hypothyroidism adversely affects Leydig cell proliferation, differentiation, along with impaired steroidogenesis [71, 72].

 Hampered of testicular function may have their origins in fetal or early life as a result of abnormal development or proliferation of Sertoli cells. A compromised antioxidant defence system marked by increased protein carbonylation, disturbed redox status during neonatal hypothyroidism, might have contributed to poor growth and development of testis by affecting spermatogenesis and steroidogenesis in rats before puberty as indicated by reduced germ cell number, complete absence of round spermatids, decreased seminiferous tubule diameter, and decreased testosterone level. Such type of altered testicular physiology by hypothyroidism is reflected in adulthood with hampered fertility as evidenced by reduced total viable germ cells [25] and sperm counts [7].

## Figures and Tables

**Table 1:** Effect of neonatal PTU treatment on bodyweight (g) of animals. Data are expressed as mean ± S.D. of 5 observations and subjected to unpaired Student's *t*-test. Statistical significance was accepted at *P* < 0.05. Control and hypothyroid groups were found to differ significantly at  *P* < 0.05 as represented by superscripts of different letters. Group-I (control): 30-day-old control rats; Group-II (hypothyroid): 30-day-old rats with PTU treatment from day 1 postpartum to day 30 postpartum.

		Group-I (control)	Group-II (hypothyroid)
Bodyweight (g)	Initial (on date of birth)	6.37 ± 0.28^a^	6.69 ± 0.44^a^
	Final (on date of sacrifice)	55.00 ± 6.85^a^	28.00 ± 4.47^b^
Weight gain (g)		48.63 ± 6.68^a^	21.31 ± 4.61^b^
% Decrease in body weight from control		Nil^a^	42.42 ± 9.20^b^

**Table 2:** Effect of neonatal PTU treatment on testis and accessory sex organs (g/100 g body weight). Data are expressed as mean ± S.D. of 5 observations and subjected to unpaired Student's *t*-test. Statistical significance was accepted at *P* < 0.05. Control and hypothyroid groups were found to differ significantly at *P* < 0.05 as represented by superscripts of different letters. Group-I (control): 30 day old control rats; Group-II (hypothyroid): 30-day-old rats with PTU treatment from day 1 postpartum to day 30 postpartum.

		Group-I (control)	Group-II (hypothyroid)
Weight (g/100 g body weight)	Testis	0.92 ± 0.09^a^	0.28 ± 0.02^b^
	Epididymis	0.208 ± 0.01^a^	0.207 ± 0.02^a^
	Seminal vesicle	0.094 ± 0.01^a^	0.059 ± 0.005^b^
	Ventral prostate	0.103 ± 0.012^a^	0.08 ± 0.006^b^

**Table 3:** Effect of neonatal PTU treatment on serum total T_3_, total T_4_, testosterone levels (ng/mL), and thyroid stimulating hormone (TSH) level in *μ*IU/mL). Data are expressed as mean ± S.D. of 5 observations and subjected to unpaired Student's *t*-test. Statistical significance was accepted at *P* < 0.05. Control and hypothyroid groups were found to differ significantly at *P* < 0.05 as represented by superscripts of different letters. Group-I (control): 30-day-old control rats; Group-II (hypothyroid): 30-day-old rats with PTU treatment from day 1 postpartum to day 30 postpartum.

Serum hormones	Group-I (control)	Group-II (hypothyroid)
Total T_3_ (ng/mL)	1.68 **± **0.16^a^	1.02 **±** 0.08^b^
Total T_4_ (ng/mL)	13.00 **± **3.92^a^	3.00 **± **0.61^b^
TSH (*μ*IU/mL)	0.12 **±** 0.027^a^	1.69 **± **0.089^b^
Testosterone (ng/mL)	0.25 **±** 0.03^a^	0.09 **± **0.02^b^

**Table 4:** Effect of neonatal PTU treatment on germ cell count (number/tubule) and seminiferous tubule diameter (expressed in *μ*) in Stage-VII seminiferous tubules of testes. Data are expressed as mean ± S.D. of 5 observations from five different animals and subjected to unpaired Student's *t*-test. Each observation per animal is the mean of 30 observations. Statistical significance was accepted at *P* < 0.05. Control and hypothyroid groups were found to differ significantly at *P* < 0.05 as represented by superscripts of different letters. Group-I (control): 30-day-old control rats; Group-II (hypothyroid): 30-day-old rats with PTU treatment from day 1 postpartum to day 30 postpartum.

	Group-I (control)	Group-II (hypothyroid)
Primary spermatogonia (number/tubule)	25.12 ± 0.41^a^	19.11 ± 0.17^b^
Number of spermatocytes (number/tubule)	15.77 ± 1.65^a^	3.31 ± 0.33^b^
Round spermatids (number/tubule)	46.50 ± 5.02^a^	Nil^b^
Sertoli cell number (number/tubule)	30.09 ± 0.34^a^	39.05 ± 2.14^b^
Seminiferous tubule diameter (*μ*)	121.13 ± 3.98^a^	89.04 ± 3.82^b^

**Table 5:** Effect of neonatal PTU treatment on total, oxidized, reduced glutathione contents (nmol thiols/g tissue) of testes of rats. Data are expressed as mean ± S.D. of 5 observations and subjected to unpaired Student's *t*-test. Statistical significance was accepted at *P* < 0.05. Control and hypothyroid groups were found to differ significantly at *P* < 0.05 as represented by superscripts of different letters. Group-I (Control): 30 day old control rats; Group-II (Hypothyroid): 30-day-old rats with PTU treatment from day 1 postpartum to day 30 postpartum; T-GSH-eq: total GSH equivalent; GSH: reduced glutathione, GSSG: oxidized glutathione.

		Group-I (control)	Group-II (hypothyroid)
MF	T-GSH-eq (nmol thiols/g tissue)	108.89 ± 8.53^a^	54.45 ± 4.26^b^
	GSSG (nmol thiols/g tissue)	18.59 ± 1.53^a^	15.49 ± 1.28^b^
	GSH (nmol thiols/g tissue)	71.72 ± 5.48^a^	23.47 ± 1.73^b^
	GSH : GSSG (nmol thiols/g tissue)	3.86 ± 0.04^a^	1.52 ± 0.02^b^

PMF	T-GSH-eq (nmol thiols/g tissue)	1227.23 ± 96.12^a^	1133.54 ± 80.64^a^
	GSSG (nmol thiols/g tissue)	116.98 ± 9.81^a^	89.43 ± 8.21^b^
	GSH (nmol thiols/g tissue)	992.74 ± 76.71^a^	954.40 ± 79.96^a^
	GSH : GSSG (nmol thiols/g tissue)	8.48 ± 0.07^a^	10.75 ± 1.40^b^
